# Are Animals Immortal?

**Published:** 1893-02

**Authors:** B. F. Underwood


### ARE ANIMALS IMMORTAL?

B. F. UNDERWOOD, in the "WORLD'S ADVANCE THOUGHT."

Animals may say: We have fundamentally the same natures that you have. We feel pleasure and pain, and are subject to moods; we have affection, jealousy, vanity, and pride; we enjoy the smile of approval from our superiors, and dread their displeasure; we are not devoid of imitation and curiosity. We have some sense of beauty, some imagination, and some power of reasoning. We are not entirely destitute of reverence. We are capable of improvement by education and inheritance. Your philosophers teach that mind is imperishable. Certainly, we have minds, distinct individual minds. Mental as well as bodily characteristics are subject to the law of heredity with us, precisely as they are among human beings. If your minds are immortal, why are not our minds also immortal? Your philosophers refer, in proof of man's immortality, to the fact that his consciousness persists, while the atoms of his brain and body are constantly changing, that memory and identity extend throughout years, although the body has changed many times, showing that the impressions must be made on something that is not, like the brain, subject to change. This is just as true of us. The atoms come and go; but our identity, as shown in memory, reaching back a dozen years and more, persists amid all material fluctuations. Your Darwins and Haeckels and Wallaces have shown what your observation should have taught you, that you are derived from the lower animals—the lower animals we say because you yourselves are animals. Go far enough back, and your ancestors and ours were the same creatures. Since our origin is the same, must not our nature and destiny be the same? Your bodies have been developed from animal bodies, your minds from animal minds. If, then, your minds are immortal, ours must be; for how could a being who is indestructible and immortal have evolved from a perishable being? To say that the capacity for immortality was somewhere acquired during the process of evolution from apehood to manhood, is to make use of an unsupported assumption, opposed to continuity, the primary fact of evolution, in order to enable you to deny our immortality and assert your own, There is another consideration we may mention in our behalf. Your theologians say that a future life is necessary to prevent the ultimate defeat of justice, since it often fails here. Think of the millions of animals that have been hunted for sport, beaten, tortured and wantonly killed—often, too, by men they were serving with all their strength and the best they knew. Where is the justice of a God who would confer immortality upon all who have found their chief sport in tormenting and destroying animals, and give the animals no recompense for their sufferings, extending through long dreary centuries, in the aggregate beyond the power of computation, and in horribleness beyond the power of Hogarth's pencil to describe? Justice requires that we have a future life. Moreover from the first, man has been surrounded by animals; they have been his companions, and they are indispensable to his happiness. He keeps them now, even when they are of no utility to him; and in the city parks are kept deer, swans, and birds of song for the pleasure of the people. In the past, men were generous enough to believe that we would share with them the future; and, even now, the Indian of the plains:

> Thinks, admitted to that equal sky,
> His faithful dog shall bear him company.

One of your own poets, while speaking our praises, bears testimony to the indispensableness of our presence and our companionship to man's happiness—an indication that, if man is immortal, we, too, are immortal:

> I think I could turn and live with animals, they are so placid and self-contained;
> I stand and look at them sometimes for an hour at a stretch;
> They do not sweat and whine about their condition;
> They do not lay awake in the dark and weep for their sins;
> They do not make me sick discussing their duty to God;
> Not one is dissatisfied—not one is demented with the mania of owning things:
> Not one kneels to another, nor to his kind that lived thousands of years ago.

